# New era: prospects for managing cancer of unknown primary

**DOI:** 10.20892/j.issn.2095-3941.2023.0168

**Published:** 2023-08-26

**Authors:** Yize Li, Yinmiao Bai, Hongchen Ji, Zhihui Liu, Hongmei Zhang

**Affiliations:** Department of Clinical Oncology, Xijing Hospital, The Fourth Military Medical University, Xi’an 710032, China

Cancer of unknown primary (CUP) is a highly heterogeneous tumor type that is confirmed to be metastatic through pathological examination; however, its primary lesion cannot be determined on the basis of detailed clinical information and diagnostic methods. The current guidelines have not changed the basic definition of CUP. Some studies have reported a decline in the incidence of CUP, to approximately 2%^1^, with the development of diagnostic techniques such as radiology, histopathology, and genetic testing. The lack of detection of primary lesions in patients with CUP may be due to the limited sensitivity of imaging technology in detecting very small tumors, the possibility that the primary tumor has regressed or remained dormant, or the presence of cells with stem cell attributes^2^. Limited evidence has suggested that alcohol consumption, diabetes, and a family history of cancer are associated with an elevated risk of CUP. However, epidemiological evidence remains insufficient to conclude that CUP has a specific risk factor profile. The natural course of CUP differs from that of tumors with clear primary lesions. CUP has the following clinical characteristics: strong invasiveness, early metastasis, symptoms and signs associated with the site of metastasis within a short time period, and an unpredictable mode of metastasis (location of metastasis different from that of the known primary tumor). The pathogenesis and pathological process of CUP are relatively complex, and the relevant theoretical models remain under exploration. The biological events leading to the undetermined primary site of metastatic cancer remain a mystery. Chromosomal abnormalities, gene expression, microvascular density, and immune microenvironmental changes in CUP have not been found to be specific^3^. Clinical studies on CUP have focused on the development of molecular diagnostics to facilitate accurate prediction of the primary site as well as specific treatments, rather than investigating current chemotherapy agents.

## Diagnosis

**Figure 1:**
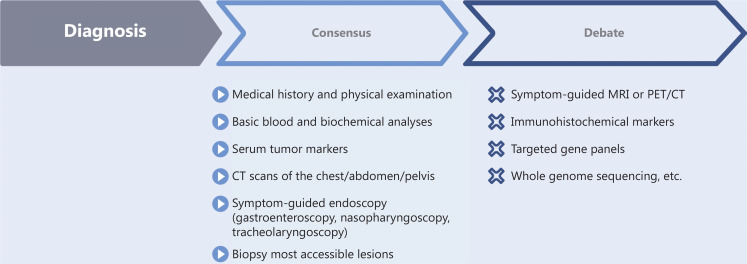
Progress in the diagnosis of CUP.

In recent years, a consensus has been reached regarding first-line CUP diagnostics, which include medical history and physical examination; basic blood and biochemical analyses; serum tumor markers; computed tomography (CT) scans of the chest/abdomen/pelvis; and accessible lesion biopsy followed by pathological diagnosis. In contrast, symptom-guided magnetic resonance imaging (MRI) or positron emission tomography/computed tomography (PET/CT) scanning, targeted gene panels, immunohistochemical markers, and whole genome sequencing remain controversial diagnostic methods (**Figure 1**). The possibility of identifying the primary tumor through further diagnosis must be weighed against the burden of testing and the delay in starting treatment.

### Immunohistochemistry

Pathology and immunohistochemistry (IHC) detection remain the “gold standards” for CUP diagnosis. IHC is an important tool for determining the tumor type, subtype, and site of origin, and it can be used to evaluate specific protein expression in tissue samples to confirm the cancer type. A consensus has been reached regarding determination of the origin of tumor tissue through multiple rounds of IHC tests, such as lineage specific and organ specific tests, and the importance of comprehensive and structured immunohistology has been emphasized. On this basis, additional IHC markers are detected according to the relevant differential diagnosis. The European Society for Medical Oncology (ESMO) guidelines in 2022 newly included decision tree algorithms for the differential diagnosis of TTF1-negative non-small cell lung cancer (NSCLC), TTF1-positive NSCLC, intrahepatic cholangiocarcinoma, ovarian cancer, renal cell carcinoma, salivary gland carcinoma, and breast cancer. The process considers the immunohistology, location and image morphology, local lymph nodes, and further metastasis patterns exhibited by the tumor under consideration as a potential primary tumor^4^. However, light microscopy combined with IHC can determine the tissue origin of only approximately 30% of CUP cases^5^, and no single pathological marker currently enables conclusive diagnosis. Importantly, CUP guidelines, such as those from the Dutch Oncoline, ESMO, National Comprehensive Cancer Network (NCCN), National Institute of Health and Care Excellence (NICE), Spanish Society of Medical Oncology (SEOM), and China Anti-Cancer Association (CACA), contain numerous differences in the recommended specific IHC markers^6^. Therefore, clinicians must further determine which IHC markers to use according to geographical differences in tumor incidence and other clinical findings.

### Tumor markers

Serum tumor markers can help clinicians analyze the disease; monitor efficacy, recurrence, and metastasis; and predict prognosis. However, multiple tumor markers are simultaneously elevated in a non-specific manner in nearly 70% of patients with CUP^7^, and specific tumor markers with prognostic and/or predictive value remain lacking. Beyond AFP (hepatocellular carcinoma), PSA (prostate cancer), and CA125 (ovarian cancer), which have relatively specific diagnostic value, other tumor markers must be comprehensively analyzed according to other clinical information from patients.

### Imaging

The importance of PET/CT in imaging diagnosis has been debated. Nonetheless, PET/CT has been found to accurately describe the true extent of disease and identify lesions that are difficult to detect with other imaging tests. Therefore, many studies have used PET/CT scans to assess the sensitivity, specificity, and detection rate of primary tumors in patients with CUP. Recent studies have shown that the success rate of PET/CT in detecting origin has increased from 66% to 87%, and as many as one-third of patients undergo a sequential shift in management strategy as a result of identification of the primary tumor^8,9^. Although statistics suggest that PET/CT is a better tool for identifying cancer origins than MRI (22%–44% *vs.* 20%–27%)^10^, it has drawbacks, such as poor sensitivity to smaller lesions, considerable false-positive and false-negative results, and unproven cost-effectiveness of use as a standard treatment. Therefore, more studies are needed to evaluate patient survival rates after the application of PET/CT for CUP. In addition, some tracers, such as ^68^Ga-DOTATOC PET/CT for neuroendocrine tumors, ^68^Ga-PSMA PET/CT for prostate cancer, and ^18^F-FES PET/CT for breast cancer, must be further validated for clinical use.

### Molecular biology

The rapid development of molecular detection has greatly facilitated the diagnosis of CUP. These methods do not replace traditional histopathological analysis but instead enhance the traditional detection, thereby providing more clues for the diagnosis of CUP. In clinicopathologically unresolved CUPs, mutations and mutational signatures have been found to provide additional diagnostic evidence in 31% of cases, whereas GEP classification is useful in only 13% of cases, and oncoviral detection is useful in 4% of cases^11^. In patients with CUP whose tissue specimens are limited, prioritizing genomic testing to guide additional further diagnosis may provide more information than using an expanded IHC panel. When tumor tissue is not available, liquid biopsies on circulating tumor cells and ctDNA from the blood can also provide a useful source of molecular information. In addition, researchers have combined pathology, genetic testing, and artificial intelligence (AI) technologies to mine information from patients’ clinical and genetic testing data by using deep-learning algorithms, and consequently predict tumor origin in patients. In the future, AI models must be applied to improve the diagnosis of CUP, and clinical trials will be necessary to determine whether these models can improve diagnostic ability and patient prognosis^12^.

## Treatment

The basic division between CUP with favorable *vs.* unfavorable prognosis still exists in the guidelines. The subgroup with favorable prognosis presents with local metastases or obvious analogies to certain cancers with a known primary, and is found in approximately 15%–20% of CUP cases. The prognosis of these patients is improved by radical resection of isolated or localized lesions, combined with timely radiotherapy and chemotherapy. The remaining patients have poor prognosis and are usually treated with empiric chemotherapy, but their median overall survival is less than 1 year. Clinical trials of chemotherapy for CUP have primarily explored the efficacy of empirical chemotherapy, including platinum, taxane, gemcitabine, vinca alkaloids, and irinotecan. However, no evidence indicates that any modality has statistically significant superior efficacy. Overall, the combination of platinum with taxanes or gemcitabine is widely accepted as the gold standard of treatment.

**Figure 2:**
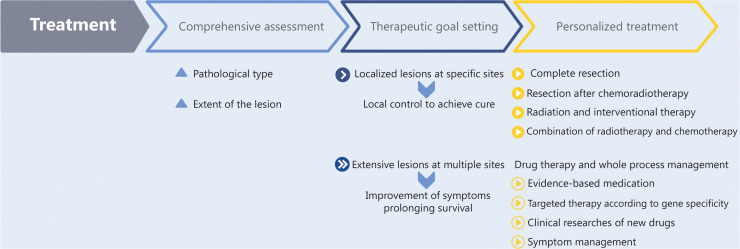
Hierarchical management and precision treatment of CUP (CACA).

The CACA CUP guidelines suggest hierarchical management and precision treatment (**Figure 2**). The recommended treatment procedures are as follows: (1) Comprehensive assessment, involving evaluation of the pathological type of the patient (adenocarcinoma, squamous cell carcinoma, neuroendocrine tumor, germ cell tumor, sarcoma, and other types) and the extent of the lesion (specific sites and limited lesions, or multiple sites and extensive lesions). (2) Therapeutic goal setting, involving local control to achieve cure or amelioration of symptoms, and prolonged survival. (3) Personalized treatment, (a) radical treatment should be the goal for patients with localized lesions at specific sites: for single lesions or a limited number of independent lesions, complete resection is recommended; for single large lesions, single lesions with clear external invasion, or limited multi-lesion fusions, resection after chemoradiotherapy is recommended; for inoperable superficial lesions or deep lesions, such as those in the liver, lung, or brain, radiation and interventional therapy can be considered; reasonable combinations of radiotherapy and chemotherapy, as well as adjuvant chemotherapy after surgery or interventional therapy. (b) For patients with extensive lesions at multiple sites: drug therapy should be the main treatment, and whole process management should be performed, including evidence-based medication according to pathological types; targeted therapy according to gene specificity; clinical studies of new drugs; and symptom management, including pain relief, nutrition, psychology, moderate exercise, or traditional Chinese medicine.

### Specific treatment

Molecular classification tools are gradually maturing, and techniques such as gene expression profiling, epigenomics, microRNA analysis, and liquid biopsy have been applied in the diagnosis of primary sites of CUP, thus bringing hope for the development of specific treatments for CUP. In contrast to the non-specificity of traditional chemotherapy, specific treatments for CUP have become a major focus in CUP clinical research, including organ-specific treatment based on tumor tissue origin gene detection, and target-specific treatment based on NGS. However, determination of the origin of tumor tissue has not been demonstrated to further optimize treatment and prolong survival in CUP^13–15^. A meta-analysis has found that patients with CUP receiving organ-specific chemotherapy tend to benefit from empirical chemotherapy, but the difference was not statistically significant (*P* = 0.06)^16,17^, thus implying that estimation of the primary site according to the metastatic pattern, IHC histological findings, and genetic mutation and molecular analysis might have minimal effects on patient survival. The main reasons for this lack of influence might be the accuracy of primary disease tracking methods; differences in populations and intervention methods among clinical trials; and the lack of large-scale randomized prospective clinical trials. Therefore, specific treatment is not a routine clinical recommendation in the NCCN, ESMO, CACA, and other guidelines. The first prospective randomized controlled phase III clinical study (NCT03278600) evaluating the efficacy of specific treatments for patients with CUP according to tumor tissue origin is underway. If the results are positive, the standard of care for CUP with unfavorable prognosis is expected to substantially change, and new revisions to the guidelines are likely to be warranted.

### Molecular targeted therapy

In a comprehensive study of 200 CUP specimens, the use of a hybrid-capture-based NGS assay has enabled the identification of at least 1 potentially targetable genomic alteration in 85% of the CUP specimens^18^. Genomic alterations (including variants of unknown significance) have been found in approximately 90% of blood-derived cell-free DNA samples from evaluable patients with CUP (*n* = 1,931)^19^. However, high frequency instructive targets for CUP have been reported. Whether target-specific therapy based on NGS testing would benefit patients with CUP remains unclear. Given that tissue- and blood-based NGS testing can provide a potentially effective treatment option for patients with CUP, the guidelines still advocate NGS testing for tumor tissue, and, when tumor tissue is not available, liquid biopsies can be performed on circulating tumor cells and ctDNA from patient blood. No targeted drugs for the treatment of CUP are currently approved. However, a series of pancancer-targeted drugs based on specific genetic changes have been approved, thus overcoming the limitations of tumor location and promising the start of a new chapter in the anti-tumor treatment of CUP. Crucially, the efficacy of targeted drugs widely varies across cancer types, and the value of these drugs in CUP if the tissue origin is not identified is uncertain. The most striking example of this phenomenon is the varying responses of different tumor types to BRAF inhibitors, ranging from high response in BRAF V600E mutated melanoma to a complete lack of activity in BRAF V600E mutated colorectal cancer. To date, the documented response of patients with CUP to targeted therapies has been limited to anecdotal descriptions of case reports. Similarly, evidence from several basket trials examining advanced metastatic cancer, including a subset of patients with CUP is weak.

### Immunotherapy

In 2022, pembrolizumab was first reported to exhibit good antitumor activity in phase II clinical studies of patients with advanced CUP. In addition, results from a multicenter phase II study (NivoCUP) have indicated that nivolumab has elevated clinical efficacy in CUP with high expression of programmed death ligand 1 (PD-L1), high tumor mutational burden, and high microsatellite instability. Thus, CUP is an immune hot tumor, and biomarkers for predicting immune efficacy can indicate the benefits of immunotherapy and further improve treatment efficacy. In contrast, on the basis of the estimated origin of the tissue, no significant difference in efficacy has been observed among tumor subgroups^20^. Therefore, immunotherapy may be a future option for patients with CUP with poor prognosis. At present, several related clinical studies are in progress, which are expected to provide further evidence of the immunotherapy and efficacy evaluation of CUP.

## Perspective

Increasing knowledge of cancer biology enables more accurate classification, diagnosis, and prognosis assessment, and provides guidance for tailoring specific treatments. However, the data for CUP are largely preliminary. CUP is a group of heterogeneous metastatic tumors with complex biological characteristics, whose heterogeneity is determined by many factors, such as genetics, the transcriptome, and the microenvironment. In the future, comprehensive analysis of CUP, incorporating genomics, transcriptomics, epigenomics, proteomics, and immunomics, will facilitate multi-level and overall understanding of the pathogenesis of CUP, and consequently provide basic understanding to guide clinical practice.

A basic consensus has been reached regarding the multi-layer examination strategy, the initial diagnostic examination, and the expected time frame for diagnosis for CUP. However, the clinical use of follow-up complementary tests as well as advanced diagnostic techniques such as symptom-guided MRI or ultrasound, PET/CT scans, additional IHC testing, targeted gene testing, and whole genome sequencing in diagnostic tests (complementary or advanced) remains debated. The goal of future research in CUP diagnosis should be aimed at reaching an international consensus regarding classification based on CUP diagnostic techniques. The standardization of diagnostic methods will enable international comparisons of incidence, treatment, and survival among patients with CUP. Consequently, additional clinical studies could be performed, and the treatment and survival of patients with CUP could ultimately be improved.

In CUP therapy, greater attention must be paid to the exploration of the patient population benefitting from treatment (chemotherapy, targeting, or immunotherapy) according to tumor tracing or the gene mutation spectrum. Because CUP is characterized by high heterogeneity, dynamic evolution, and a complex internal and environment, multidisciplinary teams must develop individualized treatment plans for patients to achieve optimal treatment effects. Given the increasing importance of molecular diagnostics in CUP evaluation, adding a molecular biologist to the team might seem logical. In addition, interdisciplinary cooperation is required, which is important for basic research, clinical research, and the prevention and treatment of CUP. As advances are made in large-scale clinical studies and related work, the pattern of empirical medication of CUP is expected to fundamentally change, and an evidence-based system for CUP diagnosis and treatment is expected to bring better clinical benefits to patients.
